# Shifting Listening Niches: Effects of the COVID-19 Pandemic

**DOI:** 10.3389/fpsyg.2021.648413

**Published:** 2021-04-26

**Authors:** Emily Rose Hurwitz, Carol Lynne Krumhansl

**Affiliations:** Music Cognition Laboratory, Department of Psychology, Cornell University, Ithaca, NY, United States

**Keywords:** music psychology, music listening, listening niche, music and memory, music and emotion, COVID-19, pandemic music, signature songs

## Abstract

The term “listening niche” refers to the contexts in which people listen to music including what music they are listening to, with whom, when, where, and with what media. The first experiment investigates undergraduate students’ music listening niches in the initial COVID-19 lockdown period, 4 weeks immediately after the campus shut down abruptly. The second experiment explores how returning to a hybrid semester, the “new normal,” further affected these listening habits. In both experiments, the participants provided a list of their most frequently listened-to songs during the respective period of time. From these, they identified one song that seemed most associated with this period, their “signature song,” and stated why this song seemed relevant. These reasons were coded on nine underlying themes. Three clusters were found to underlie the themes: (1) emotional responses (2) memory associations, and (3) discovery of new music. We identified songs and reasons for selecting them that represented the three clusters and related these to the lyrical content. Compared to before the pandemic, participants in both experiments report listening more in general and on Spotify, but there were no differences in listening between lockdown and the new normal. Whom they were listening with shifted overtime from family members to significant others and finally to other friends and roommates. These results demonstrate how students listen to and find new music that is meaningful to them during this unprecedented pandemic.

## Introduction

The present study investigated undergraduates’ changing music listening habits and the functions of music during the COVID-19 pandemic. We collected data after students were abruptly sent home in the spring of 2020 to complete courses entirely remotely as well as after the hybrid semester began in the fall with most students attending classes at the university either remotely or in person, the “new normal.” We assess changes in whom they are listening with and with what music media, with a special focus on Spotify. The study also asks participants to choose a song that they strongly associate with this period of time and the reasons for that choice. The reasons given for choosing particular songs and their lyrics are considered in light of three main lines of research on the uses of music: associations with emotional responses, autobiographical memories, and discovery of new music. We begin with a brief review of the literature in each of these areas, with particular consideration of possible effects of the pandemic.

One of the most frequently given reasons for listening to music is its emotional effects. Music is often used to evoke a specific emotion (Sloboda et al., 2001). Music can induce physical reactions associated with emotions such as chills, tears, and sweating (e.g., Sloboda, 1991; Grewe et al., 2009). Participants report that chills, in particular, reflect a moving, highly affective emotional state (Bannister, 2020). Highly pleasurable music that induces chills corresponds with increased activity in brain circuitry related to reward, pleasure, and emotion (Blood and Zatorre, 2001).

Music is useful for regulating emotions, especially those with negative affect. The model of Van den Tol and Edwards (2013) describing the process of choosing to listen to sad music proposes several interacting factors, both situational and individual. The goals are either attained, leading to successful emotion regulation, or not attained, resulting in increased negative affect. Successful emotion regulation can help listeners to re-assess emotions and contexts, often leading to feeling better. Many teenagers report listening to music incongruent with their emotions to change their current mood and some even depend on music to alter their state, citing it as a “friend” or “good drug” (McFerran and Saarikallio, 2014). On the other hand, listeners may use music not to derive positive emotions, but instead to ruminate, which is an effective strategy in finding consolation (Taruffi and Koelsch, 2014). Similarly, people may induce melancholy or nostalgic feelings (Van Goethem and Sloboda, 2011).

The COVID-19 pandemic has significantly impacted daily life through state and nationwide lockdowns, as well as through public health guidelines, such as mask-wearing, social distancing, and working from home, and has likely impacted emotional stability for many. On websites like Weibo, people talked about more negative emotion and less positive emotion compared to before the pandemic and were more concerned for their family and health (Li et al., 2020). People have been more stressed, anxious, and fearful during the pandemic and have displayed decreased mental well-being (Fofana et al., 2020; Jungmann and Witthöft, 2020). College students, for example, had poorer mood and decreased wellness behaviors (Copeland et al., 2021), and a majority reported that their stress and anxiety levels had increased during the pandemic (Son et al., 2020; Wang et al., 2020). Furthermore, the longevity of the pandemic impacts one’s emotional state, as Xie et al. (2020) found that after 30 days of social distancing measures, loneliness increased significantly. Boals and Banks (2020) suggest that during this time, it is important to engage in activities that provide reprieve from daily worries such as listening to and playing music. Music therapy has already been implemented to help clinical staff to cope with pandemic-related stress, and preliminary evidence shows that in Italian COVID-unit staff, receptive music therapy correlated with a significant decrease in tiredness, sadness, fear, and worry (Giordano et al., 2020). Both unstructured and structured musical activities can help individuals to regulate emotions and cope with stress.

Although some forms of media like watching television, videos, and movies have been associated with negative life satisfaction during the pandemic, music use has been correlated with positive life satisfaction (Krause et al., 2020). Recent research suggests that people used music more often to cope during the lockdown compared to before (Fink et al., 2021). Due to decreased mental well-being during the pandemic (e.g., Fofana et al., 2020; Jungmann and Witthöft, 2020), music-listening may be used to change or regulate one’s mood (Fink et al., 2021; Martín et al., 2021; Sachs et al., 2021). In a survey of 1,377 people from Spain, Martín et al. (2021) found that 73.8% of participants reported using music to regulate isolation-induced loneliness and negative emotions. This relief from loneliness also highlights music’s ability to act as a social surrogate and create a sense of togetherness when people cannot interact in-person with friends (Fink et al., 2021; Granot et al., 2021).

Another focus in the literature is on how music is important for the formation and retrieval of memories; for example, it is linked to identity through specific autobiographical memories (Baumgartner, 1992; Gabrielsson, 2011). Memories associated with music often evoke strong emotions like nostalgia (Schulkind et al., 1999; Janata et al., 2007), as well as induce chills (Bannister, 2020). Music-evoked autobiographical memories, or MEAMs, can be specific or general, frequently involving people, places, and events. MEAMs interact with specific listening niches, as music shared through generations aids in the formation of MEAMs (Krumhansl and Zupnick, 2013). While most of the MEAM literature involves laboratory experiments, recent work has assessed the salience and frequency of MEAMs in real-life settings. For example, Jakubowski and Ghosh (2019) asked participants to record the music they listen to throughout a week and keep a MEAM log. They found that even amid routine tasks, music evokes strong memories. Music has been noted to bring forth stronger memories than other stimuli, as in a study comparing memories evoked by faces to those evoked by music, the memories retrieved from listening to music were more vivid (Belfi et al., 2016). Memories evoked by music may be particularly salient due to people frequently listening to the same song, therefore strengthening memory encoding (Janssen et al., 2007).

Stressful and traumatic events affect the specificity of autobiographical memory retrieval. In *autobiographical memory overgenerality* (for a review, see Moore and Zoellner, 2007), individuals who have undergone traumatic events cannot retrieve specific memories as per instructions; rather, they describe general memories (Williams, 1996). Autobiographical memory alteration may be a way to avoid emotional distress and regulate negative emotions (Williams, 1996), and memory is often influenced by mood (for a review, see Kensinger, 2009). In addition, negative moods have been shown to reduce false memories (e.g., Storbeck and Clore, 2005; Kensinger and Schacter, 2006), and there are strong links between traumatic memories and strong emotions. In a study investigating autobiographical memory in individuals with PTSD, more stressful memories had greater emotional intensity than non-stressful memories, as did involuntary memories compared to voluntary memories (Rubin et al., 2008). However, these memories were not more fragmented. Similarly, when undergraduates were asked to retrieve three memories associated with panic, trauma, worry, social anxiety, and feeling content, those memories associated with panic and trauma had more imagery and emotion (Wenzel et al., 2004). Finally, researchers have warned about decreased working memory capacity as a result of stress and anxiety-induced mind-wandering during the pandemic (Boals and Banks, 2020), as well as the negative impacts of long-term social distancing on memory (Xie et al., 2020).

Finally, the widespread availability of streaming services allows users to easily listen to music. Currently, Spotify has over 60 million songs (Spotify, 2020) and Apple Music has over 70 million songs (Apple Music, 2020). There are three types of playlists on Spotify: user-generated playlists, Spotify-curated mood/genre playlists, and Spotify-generated user-personalized playlists. Thirty six percent of Spotify listening is spent listening to user-generated playlists, compared to 15% on Spotify-curated playlists and 17% on Spotify-personalized playlists (Spotify Technology S.A., 2018). Through Spotify-personalized playlists, targeted recommendations help listeners to find music, that is new to them but feels familiar (Luck, 2016). Luck notes that it also allows listeners to access music associated with nostalgia. Spotify playlists for each user can be broadly categorized as those for discovering new music and those for rehearing old favorites.

To assess the effect of the pandemic on streaming service usage, Sim et al. (2020) analyzed Spotify’s weekly top 200 songs for 2 years in 60 countries. They estimate that music streaming initially decreased 12.5% at the onset of the pandemic, contrary to the expectation that changes, such as working at home, would increase streaming service usage. However, Spotify use has subsequently recovered in countries that reopened. They note that in May 2020, Spotify introduced a feature called as “Listening Together,” where up to four people can simultaneously listen to their common playlists. Another innovation is listening parties, where participants (often including some of the musicians on the original album) begin listening to the same classic album at the same time and interact simultaneously on Twitter. They suggest that the stress of the pandemic has spurred people to engage in collective nostalgia (Lee and Kao, 2020).

Music serves different purposes depending on the context. Across one’s lifespan, the contexts in which a person listens to music change (Hargreaves and North, 1999). Both age and life goals influence an individual’s music (North and Hird, 2020). Thus, as an individual ages and develops different personal goals, their musical preferences and habits change in parallel. For example, individuals may have a narrow music taste during their teenage years but then broaden their preferences as they grow older (Schubert, 2016). For many teenagers, music is a means for self-actualization and the fulfillment of emotional and social needs (Tarrant et al., 2000). With age, there is also a regular progression in whom they are listening with, from parents and siblings, to friends, significant others, and children (Krumhansl, 2017). There is also a regular progression in the medium used for listening to music with the newest technologies adopted during late adolescence (Krumhansl, 2017). The listening context, together with other autobiographical memories, emotional responses, and the music itself, were collectively called the “listening niche” (Krumhansl, 2017). Given the rapid changes in context that college students have experienced during the pandemic, the present study seeks to trace the attendant changes in music listening.

## Materials and Methods

### Experiment 1

Classes at Cornell University were paused for 3 weeks on March 13, 2020. The administration urged students to return to their permanent residences. The majority of students returned to their permanent homes, and international students either had to leave the country or find accommodations within the United States to retain student visas. Classes resumed as online classes on April 06, 2020. State-wide lockdown restrictions and public health measures, such as mask-wearing, were enforced during this time.

#### Participants

One hundred and thirty-three Cornell University undergraduate students (37 males, 90 females, 6 non-binary, mean age = 20.3, and range 18–33) who were active Spotify users participated in this experiment. On average, they had been using Spotify for 4.4 years (range 0.18–9 years), and in general listened to music on Spotify on average 19.5 h/week (range 1.5–100 h/week). As this was a study of popular music, no questions were asked about formal musical training. A minority of participants (30.8%) stayed in Ithaca during lockdown, while the remainder was elsewhere, usually at home with family. Of those who had left Ithaca, they reported having left an average of 29.5 days (range 4–42 days) prior to the time they took the survey. Thus, most of them left soon after the campus shut down. This experiment was approved by the university’s Institutional Review Board.

#### Procedure

Participants filled out a Qualtrics survey that opened on April 10, 2020, exactly 4 weeks after Cornell canceled in-person classes for the rest of the semester, and it remained open for 2 weeks, closing on April 24, 2020. The survey asked participants to report their most listened-to songs from the previous 4 weeks, 6 months, and of all time. Because the survey opened exactly 4 weeks after Cornell canceled classes and quarantine began, the association songs from the 4-week category came specifically from this time period. The study then asked participants to choose a song from the most listened-to songs from the previous 4 weeks that they strongly associate with this period of time and give the reason for that choice. Unlike other studies that allowed users to select from a list of reasons for listening to music during the pandemic (e.g., Sachs et al., 2021), we intentionally chose to leave this question open-ended to explore what may be a broader range of responses. Participants then filled out demographic information. They reported their age, the ages of their parent(s), gender, where they are from originally, where they are now, and if they are currently away from Cornell, how long they have been there. They self-reported how many hours per week they listen to Spotify, how long they have used Spotify, what Spotify-generated features they regularly use, and pandemic-specific listening habits.

The pandemic-specific questions were:

How has the move to virtual class/being sent home affected how much you listen to music … – in general/on Spotify/on the radio/on streaming services other than Spotify (0 = listening a lot less, 100 = listening a lot more, and 50 = listening the same)?
Since Cornell has canceled classes and moved online, what percent of the time do you listen to Spotify … (These do not have to add up to 100%) – alone/with parents/with siblings/with significant other/with roommates/with friends other than roommates (percent)?
How do you think the songs you have been listening to for the last 4 weeks differ from those you were listening to before, if at all (open ended)?
Do some artists seem particularly relevant right now?/Why do they seem relevant (open-ended)?
Go back and look at the songs you have been listening to the last 4 weeks. Name one song that you think you will strongly associate with this period of time/Why do you strongly associate this song with this period of time (open-ended)?

### Experiment 2

The university decided to bring students back to campus for a hybrid fall semester, with some classes in-person but the majority online. While most state-wide lockdown restrictions on businesses had been lifted by the beginning of the semester, mask-wearing and social-distancing were still enforced. Students signed a behavioral compact stating that they would follow all state‐ and university-mandated pandemic guidelines. Most students came back to campus and settled into a “new normal” routine, but with many online classes and a lack of social opportunities, the semester was far from a normal semester.

#### Participants

One hundred and thirty-seven Cornell University undergraduate students (36 males, 98 females, 3 non-binary, mean age = 19.76, and range = 17–23) participated in this experiment. In addition to the active Spotify user requirement, we only recruited students who had experienced Cornell’s Spring 2020 shutdown of classes. Of the participants in Experiment 2, 16.8% had participated in Experiment 1. On average, participants had been using Spotify for 4.3 years (range 0–14 years), and, in general, listened to music on Spotify on average 15.9 h/week (range 0–88 h/week). The majority of participants (81.8%) in Experiment 2 were in Ithaca. Of those, 36.2% were living in dormitories, and 63.8% were living off campus (in apartments, sororities, or fraternities). This experiment was approved by the university’s Institutional Review Board.

#### Procedure

Participants filled out a Qualtrics survey that opened on September 30, 2020, exactly 4 weeks after Cornell began the hybrid Fall 2020 semester, and it remained open for 2 weeks, closing on October 14, 2020. Because the survey opened exactly 4 weeks after Cornell canceled classes and quarantine began, the association songs from the 4-week category came specifically from this time period.

The survey included all questions from Experiment 1, but three questions had post-lockdown counterparts:

How do you think the songs you have been listening to for the last 4 weeks differ from those you were listening to before the pandemic (if at all)/during the end of spring semester when classes were moved online (if at all; open-ended)?
How did the move to virtual class and being sent home during the spring semester/the start of the hybrid Fall 2020 semester affect how much you listen to music … In general/on Spotify/on the radio/on streaming services other than Spotify (0 = listening a lot less, 100 = listening a lot more, and 50 = listening the same)?
In the Spring 2020 semester after classes were moved online/in the Fall 2020 hybrid semester, what percent of the time did you listen to Spotify … (These do not have to add up to 100%) – alone/with parents/with siblings/with significant other/with roommates/with friends other than roommates (percent)?

## Results

### Overview of the Analyses

The survey data were analyzed to first characterize the listening niches of the participants in the two experiments, particularly, their use of Spotify and other music media, and with whom they were listening (or alone). We then analyzed which of the Spotify features they were using to discover new music and/or listening to old favorites. The participants chose a song from the list of their most frequently listened-to songs from the preceding 4 weeks that they felt most strongly associated with the period of time when they filled out the survey (just after shutdown and just after returning to the “new normal”), called their “signature song.” The reasons given were analyzed for frequent words and underlying themes, and these were clustered to find groupings of reasons for selecting the songs. Finally, the clustering of reasons given was used to select particularly representative songs for the clusters, and the lyrics of the songs were discussed as they related to the reasons given.

### Listening Niches in the Pandemic

Participants reported on whether they were listening more or less to music during the pandemic than before in general, on Spotify, on radio or other streaming services on a scale from 0 to 100, where 50 = listening the same. Tests were done to see if the responses deviated significantly from 50. In both experiments, participants reported listening more to music in general [*t*(1,268) = 7.36, *p* < 0.0001] and more on Spotify [*t*(1,268) = 6.54, *p* < 0.0001] during the period after shutdown than before the pandemic, and less on the radio [*t*(1,268) = −8.35, *p* < 0.0001] and other streaming services [*t*(1,268) = −2.27, *p* = 0.024]. However, a one-way analysis of variance revealed no difference in total hours spent listening to music between lockdown (Experiment 1) and the return to campus (Experiment 2), *F*(1, 192) = 2.52, *p* = 0.114.

**Figure 1:**
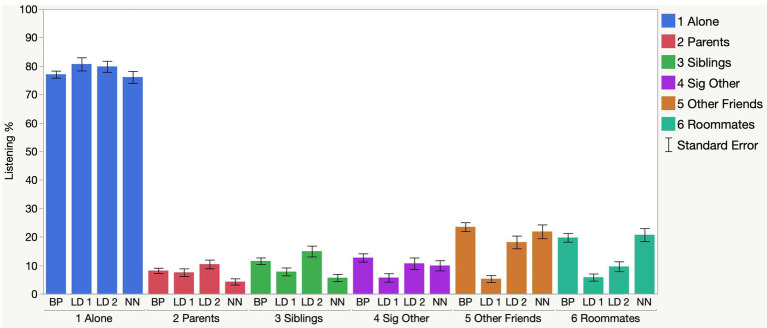
Shows the percentage of participants using Spotify alone and with others at four time periods: before pandemic (BP), in the 4 weeks after lockdown (LD 1), retrospectively, before returning to fall classes (LD 2), and after returning to classes, “new normal” (NN).

Figure 1 shows the percentage of time they were using Spotify while listening alone, or with other people, during four time periods: before the pandemic (BP, Experiments 1 and 2), right after lockdown (LD1, Experiment 1), retrospectively during lockdown after return to the classes (LD2, Experiment 2), and during the “new normal” (NN, Experiment 2). A one-way analysis of variance was done on these percentages as a function of the four time points. By far the most frequent mode of Spotify use was listening alone (average 78.1% of the time). There was no overall difference across the time periods, *F*(3,673) = 1.35, *p* = 0.259, although there was a trend to use Spotify more during lockdown at home during the summer than before the pandemic or after the return to classes. They used Spotify most with parents and siblings in the period before returning to classes (LD2) and least after returning to classes (NN), *F*(3,673) = 3.77, *p* = 0.011, *η*^2^ = 0.017 and *F*(3,673) = 6.96, *p* < 0.0001, *η*^2^ = 0.030 for parents and siblings, respectively. They generally used Spotify with significant others least right after lockdown (LD1), *F*(3,673) = 3.05, *p* = 0.028, *η*^2^ = 0.013. Finally, they reached general pre-pandemic levels of using Spotify with other friends and roommates during the new normal, *F*(3,673) = 17.37, *p* < 0.0001, *η*^2^ = 0.072 and *F*(3,673) = 16.87, *p* < 0.0001, *η*^2^ = 0.070, respectively.

### Rehearing Favorite Music and Discovering New Music

Given the prevalent use of Spotify during the pandemic, analyses were conducted to determine which Spotify features were being used to discover new music. Table 1 shows the percent of participants in each experiment who reported using these features. A clustering analysis grouped the first three, Discover Weekly, Release Radar, and Tastebreakers into one cluster; these features all seem oriented to finding new music. The remaining five, Your Top Songs [X] Year, The Ones That Got Away, Your Summer Rewind, Repeat, and Rewind, On Repeat, formed another cluster. These features seem oriented toward rehearing old favorites. This means that there were individual differences, with some participants using the discover features and others using the rehearing features.

**Table 1:** Spotify-personalized playlist features, brief descriptions, and percent of usage in each experiment.

Spotify features	Brief description	Exp. 1% users	Exp. 2% users
Discover weekly	Enjoy new music and deep cuts picked for you. Updates every Monday.	22.6	26.3
Release radar	Catch all the new music from artists you follow, plus new singles picked for you. Updates every Friday.	24.8	20.4
Tastebreakers	A playlist of songs from genres and artists you do not normally explore.	08.3^†^	02.9^†^
Your top songs [X] year	The songs you loved most this year, all wrapped up.	36.1	35.0
The ones that got away	A collection of songs you’ll wish you’d discovered earlier in the year.	09.0	05.1
Your summer rewind	A playlist featuring your old summer favorites.	17.3	18.2
Repeat and rewind	Past songs that you could not get enough of.	13.5	11.7
On repeat	The songs you cannot get enough of right now.	15.8^∗^	27.0^∗^

† *p* = 0.055;

∗ *p* < 0.05.

A one-way analysis of variance was conducted to test if there was a difference between experiments in the use of these features. There was a marginally significant decrease in the use of Tastebreakers after the return to campus, *F*(1,268) = 3.71, *p* = 0.055, *η*^2^ = 0.014 and a significant increase in the use of On Repeat, *F*(1,268) = 5.09, *p* = 0.025, *η*^2^ = 0.019. This suggests that after the return to classes, they had less time to discover new music outside of their usual genres and relied more heavily on their personal favorites. An unpublished study featuring both authors, with similar methods, was conducted in fall of 2019. The primary difference was that it did not include any questions about the pandemic. Participants were using more Spotify features after the onset of the pandemic than before, *F*(1,329) = 5.13, *p* = 0.024, *η*^2^ = 0.015.

### Selecting Signature Songs

The participants selected the song they considered to be most closely associated with the most recent 4-week period from their list of songs from that period. Of the 270 unique songs, all but four were chosen by just one person, indicating very little overlap in the songs chosen. “WAP” was chosen by four participants and “Better Days,” “Supalonely,” and “Someone to You” were each chosen by two participants.

Participants were asked why they had chosen this song in a free-response question. As in other studies that involve free response (Lonsdale and North, 2011), thematic analysis was carried out by both authors on the 270 songs to determine common patterns in reasons behind the songs’ significance. No prior assumptions were made about these reasons, and through repeated examination, nine scales emerged: whether the quarantine was mentioned explicitly, whether the song was associated with a person, a place, or an event (other than the quarantine), whether they felt nostalgia, negative, or positive emotion, or had mixed feelings when listening to the music, and whether they discovered the music during this period of time.

To give some examples of how the reasons given were coded, one reason given for choosing a song was “The song is about feeling alone, and I definitely feel very isolated during this time! The song is also very upbeat and groovy, which pulls me toward it in this difficult time.” This was dummy coded 1 on quarantine, negative, positive, and mixed emotions, and 0 on the other scales. Another example is, “Before we left the college campus, my friend and I drove around campus listening to this artist and his songs.” This was dummy coded 1 for mentioning quarantine, person, place, event, and nostalgia, and 0 on the other scales. Finally, “During this time I’ve been trying to find new music and this is my favorite song that I found recently” was dummy coded 1 on discovered during this period of time, and 0 on the other scales. The interrater reliability was assessed by correlating the two dummy codes given by the two raters on the nine scales. All the correlations were significant at *p* < 0.0001 [average *r*(131) = 90.73 for Experiment 1, *r*(135) = 94.46 for Experiment 2], and ranged from *r*(131) = 0.800 for nostalgia in Experiment 1 to *r*(135) = 1.0 for associated with place in Experiment 2.

Table 2 shows the percent of participants whose reasons were coded as present on each of the nine scales. A one-way analysis of variance was conducted to test for differences between the experiments. As can be seen, the quarantine was mentioned most frequently in Experiment 1 and more than in Experiment 2, *F*(1,268) = 81.90, *p* < 0.0001, *η*^2^ = 0.234. The only other significant difference between the experiments was that place, often the university or town, was more often referred to in Experiment 1, *F*(1,268) = 4.37, *p* = 0.038, *η*^2^ = 0.016, although there was a marginally significant decrease in mentioning songs discovered during this period, *F*(1,268) = 3.38, *p* = 0.067, *η*^2^ = 0.012.

**Table 2:** Themes underlying reasons for each signature song and percent of participants mentioning each theme.

Reason given for song	Exp. 1% participants	Exp. 2% participants
Quarantine mentioned	42.5^∗∗∗^	11.6^∗∗∗^
Associated with person	20.3	25.2
Associated with place	16.2^∗^	08.0^∗^
Associated with event	17.7	18.1
Nostalgia	18.0	13.1
Negative emotion	25.2	24.4
Positive emotion	21.8	27.0
Mixed emotion	25.6	19.3
Discovered in pandemic	35.7^†^	25.5^†^

† *p* = 0.067;

∗ *p* < 0.038;

∗∗∗ *p* < 0.0001.

**Figure 2:**
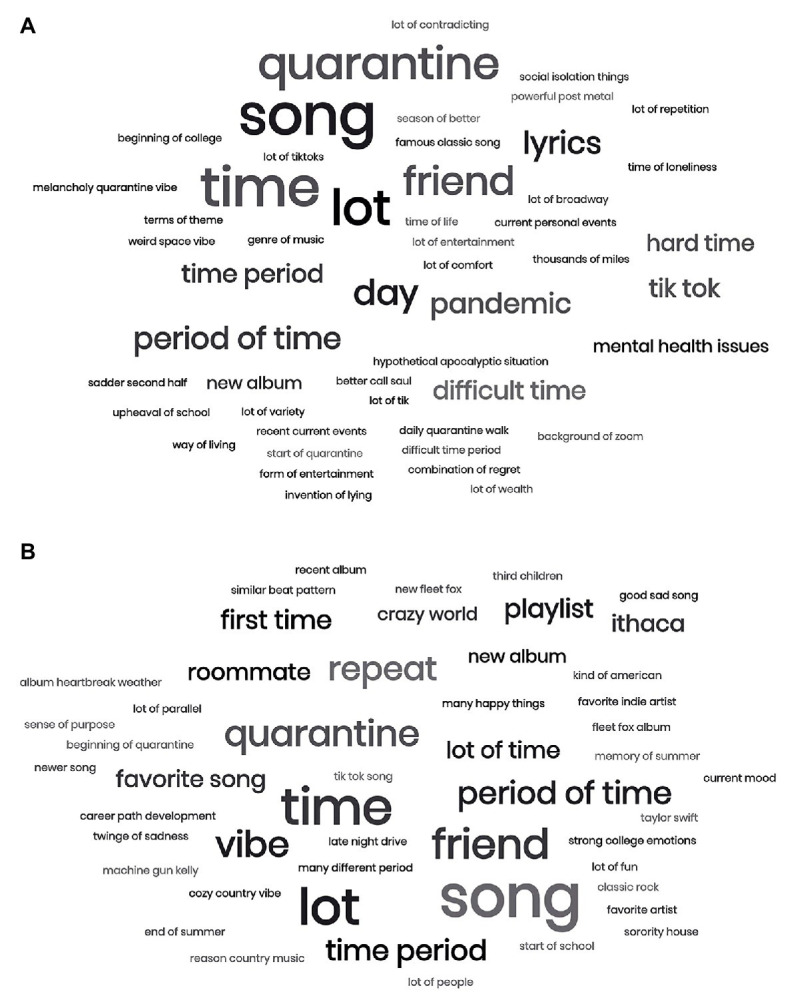
Word clouds of most frequent words/phrases from reasons behind choosing signature songs. **(A)** Experiment 1 reasons **(B)** Experiment 2 reasons.

To get a visual impression of the reasons why people were choosing their songs, we created word clouds (Figures 2A,B) of the reasons for selecting the songs using the WordCloud Generator from MonkeyLearn.^1^ This tool also generates a frequency list of words and phrases. In Experiment 1, the most frequent 10 words/phrases were “song,” “time,” “lot,” “quarantine,” “friend,” “day,” “lyrics,” “period of time,” “pandemic,” and “tik tok.” In Experiment 2, many of these words and phrases were also among the most frequent 10, reinforcing the similarities across experiments in the reasons people were listening to music. The only new words in the top 10 were “vibe,” “repeat,” “first time,” and “playlist.”

Although quarantine was not mentioned explicitly in the prompt, it may have been keyed by: “Name one song that you think you will strongly associate with this period of time.” Consequently, it was not included in the clustering analysis applied to the reasons given. The clustering grouped together negative, positive, and mixed emotions. The second cluster grouped together associated with person, place, event, and nostalgia, all of which have a memory component. Discovered during the pandemic was negatively related to the first cluster analysis, so will be considered its own cluster.

For each of the three clusters, emotion (negative, positive, and mixed), memory (person, place, event, nostalgia), and discover, a song was selected that had reasons that best matched the cluster’s members. For example, the song chosen to represent the memory cluster would be one that the participant mentioned a person, place, event, and nostalgia as a reason for choosing the song, but not the other four types of reasons. These were dummy coded and correlated with the scoring of each participant’s reasons for choosing the song on the eight scales. The “signature song” to represent each cluster was one in which the correlation was equal to 1.00.

### Analysis of Signature Songs

Table 3 shows three of the songs that represented each cluster, the reason given, and some representative lyrics that were retrieved from Genius.^2^ One representative song for the emotion cluster was “Supalonely” by BENEE. This participant expressed negative, positive, and mixed emotions in their reason for selecting this song, noting that they “feel very isolated during this time.” They resonate with the song’s sad lyrics, but it also feels “very upbeat and groovy.” The lyrics clearly express this negative outlook on life, with lines such as “I’m a sad girl, in this big world.” However, with BPM of 129 and pop melody, this song is also fast-paced and danceable. Thus, this song serves to both validate the participant’s negative feelings with lyrics and to uplift them with the overall sound.

**Table 3:** Songs examples for each cluster, the associated reasons for choosing the song, and selected lyrics.

Cluster	Song	Reason	Lyrics
Emotion	Supalonely (BENEE, 2019)	“The song is about feeling alone, and I definitely feel very isolated during this time! The song is also very upbeat and groovy, which pulls me toward it in this difficult time.”	“I’m a sad girl, in this big world It’s a mad world All of my friends Know what’s happened You’re a bad thing I know I f‐ up, I’m just a loser Shouldn’t be with ya, guess I’m a quitter While you are out there drinkin’, I’m just here thinkin’ ‘Bout where I should’ve been I’ve been lonely, mm, ah, yeah”
Memory	Chasin’ You (Wallen, 2018)	“Before we left the college campus, my friend and I drove around campus listening to this artist and his songs.”	“We used to chase that Chattanooga fray Couple a kids in a Chevrolet Catch a little air when we cross the tracks Sipping on something from a paper sack You hang your shirt on that maple lamp Slipping through the moon to the river bend Wasn’t very long I was jumping in, jumping in I guess I’m still doing now, what I was doing then Chasing you, like a shot of whiskey Burning going down, burning going down Chasing you, like those goodbye taillights Heading west to anywhere out of this no where town”
Discover	Beige (Yoke Lore, 2017)	“During this time I’ve been trying to find new music and this is my favorite song that I found recently”	“You know you are beautiful But that ain’t half the gold treasure in your soul what you got ‘cause I want it all With your fingers in my mouth, I fail to see your faults So please do not let me fall So please do not let me fall And I think we’d survive in the wild We would eat plants and roots and dream about electric fans Baby, could you kill a man? Could you look in his eyes and feel the fire drain out of his hands? Baby do you think about the past? Do you wonder if every stupid little thing has led us to this”

One of the songs that best represented the memory cluster was “Chasin’ You” by Morgan Wallen. For this participant, the song represents a music-evoked autobiographical memory of a time before the pandemic. They noted a specific time where they “drove around campus listening to this artist and his songs” with a friend. The lyrics express a similar sentiment, stating, “We used to chase that Chattanooga fray/Couple a kids in a Chevrolet.” During the initial stages of the pandemic, undergraduates were away from both campus and friends. Thus, this memory is important for the participant, as they may be missing both.

One of the songs that best represented discovery was “Beige” by Yoke Lore. This participant states they have found new music during this time, and this is their “favorite song [they] have found recently.” The lyrics are rich with imagery (“We would eat plants and roots and dream about electric fans/Baby, could you kill a man?/Could you look in his eyes and feel the fire drain out of his hands?”) but in contrast to the lyrics for the emotion cluster song, express less concrete emotion. Thus, the participant may simply like this song for its aesthetic quality, rather than for emotion or memory associations. Additionally, though this song is new to the participant, it was released 3 years prior in 2017.

**Figure 3:**
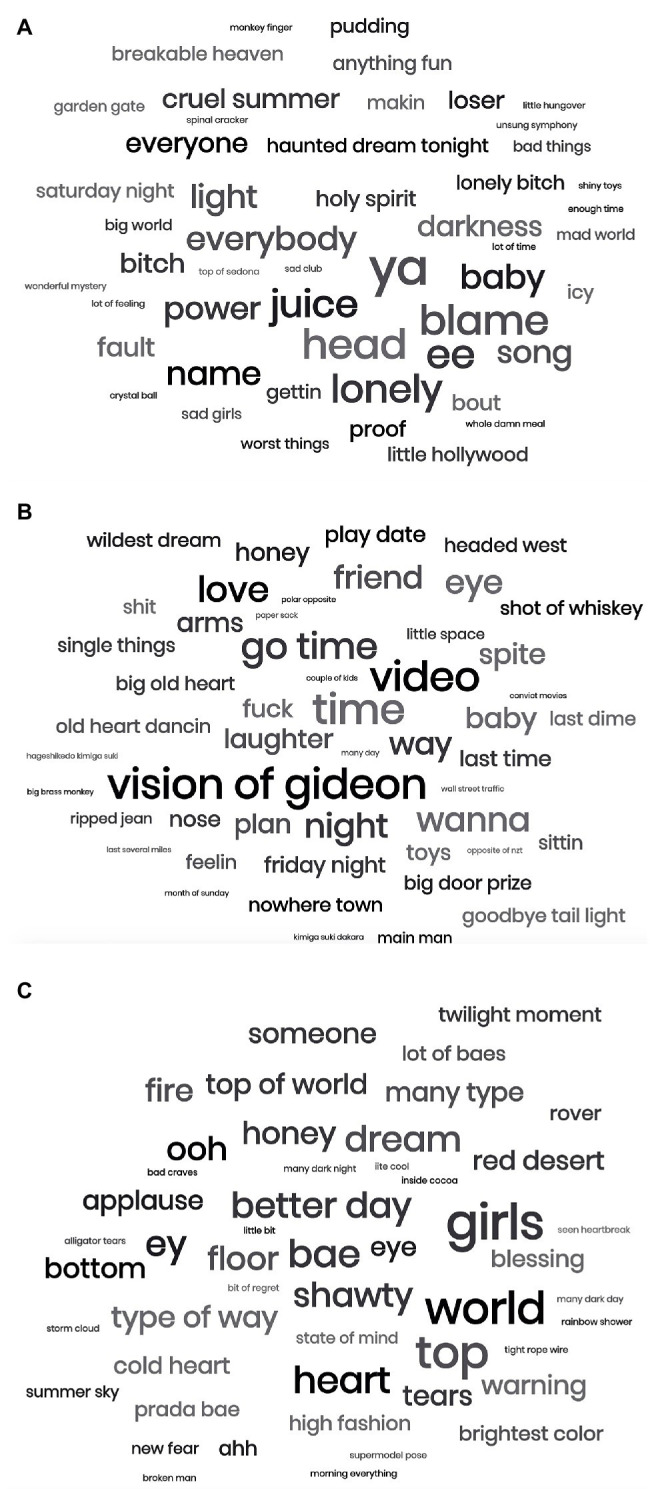
Word clouds of most frequent lyric words/phrases from top 10 songs associated with each cluster. **(A)** Emotion cluster **(B)** memory cluster, and **(C)** discovery cluster.

As another approach to understanding the semantic content of the lyrics, we also retrieved the song lyrics for the top 10 signature songs correlated with each cluster using Genius. The resulting word clouds of the most frequent words in the lyrics for each cluster are shown in Figures 3A–C. For the emotion cluster, the five most frequent words (excluding “ya” and “ee,” which are not real words, and “juice,” which is the title of a song) are “head,” “blame,” “lonely,” “baby,” and “everybody.” In addition to “lonely” and “blame,” there appear to be a prevalence of other emotion words such as “worst,” “fun,” “cruel,” “breakable,” “sad,” “mad,” and “loser.” For the memory cluster, these emotion words appeared less prevalently, but there were more mentions of time, place, and people. The five most frequent words (excluding “vision of Gideon,” which is the title of a song) were “time,” “video,” “go-time,” “love,” and “wanna,” and there were other related words such as “night,” “nowhere town,” “plan,” “Friday night,” “last time,” “friend,” and “baby.” For the discover cluster, the five most frequent words were “girls,” “world,” “top,” “bae,” and “heart.” These lyrics seem to be more general themes frequently found in pop songs, presumably songs that people are discovering during this period of time. They also seem to be more positive themes, with phrases such as “better day,” “top of world,” and “type of way.”

## Discussion

The main objective of this study was to characterize the habits and motivations for listening to music during the COVID-19 pandemic, with a special focus on Spotify. We found that by far the majority of time spent using Spotify was listening alone during all periods of time included in the two experiments. Research suggests that even solitary music listening can be an activity in which people find a sense of connection (Schäfer et al., 2020). Recent studies about music listening in the pandemic have indicated that people have turned to music to provide a sense of togetherness (Fink et al., 2021; Granot et al., 2021), as well as to decrease pandemic isolation-induced loneliness (Martín et al., 2021). Like Krause et al. (2020), we found no difference in hours spent listening to music in general across the different time points of the pandemic, as well as no difference in time spent listening to music alone across time periods.

However, when using Spotify with others, there was a regular progression in whom they were listening with. In the period after lockdown and before returning to classes, there was an increase in using Spotify with other family members, significant others (either locally or remotely), and finally returning to normal levels with other friends and roommates after the return to campus. Spotify seems to have been a useful tool for regaining normal levels of sharing music with others. This is similar to the study by Sim et al. (2020), which documented a return to normal levels of streaming as countries reopened.

Our participants’ responses provide insight into the reasons behind listening to music during the pandemic. The words most frequently used in the reasons for choosing songs in Experiment 1 relate to themes of time, friendship, quarantine, and usage of TikTok, on which many participants report discovering new music. The most frequent words in these responses in Experiment 2 were extremely similar, which reinforces the likeness of music usage during these two periods despite the loosening of lockdown restrictions during the latter. However, it is interesting to note that “repeat” and “playlist” were among the top 10 words in the signature song reasons for Experiment 2. This supports our finding that after returning to campus, participants reported using the Spotify-curated playlist “On Repeat” significantly more than in the initial lockdown period.

The reasons given for identifying a song as relevant during the pandemic fell into three clusters: memory (associated with person, place, event, or evoking nostalgia), emotion (negative, positive, and mixed), and discovered during the pandemic. These clusters correspond to three of the major themes in studies of the uses of music, as reviewed in the introduction. Thus, these general motivations for listening to music appear not to be altered significantly by the pandemic.

As many participants suggested, music can both repair mood and decrease feelings of loneliness and isolation (Schäfer et al., 2020). Many of the reasons listeners provided for choosing songs especially associated with this period of time often contained reports of mixed emotions. Music can express multiple emotions at one time (Krumhansl, 1997; Vines et al., 2005), and this is evident in the reasons given for choosing particular songs. Using music to cope and regulate emotion is a common trend in reasons for listening to music during the pandemic (Fink et al., 2021), as other studies have also highlighted music as important for mood regulation (Fink et al., 2021; Martín et al., 2021; Sachs et al., 2021) and decreasing negative feelings stemming from social distancing and isolation (Martín et al., 2021). It is interesting to recall the findings of Krause et al. (2020) who found the same beneficial effects of music on pandemic life satisfaction are not present for other forms of media such as television, videos, and movies.

The reasons given for selecting music that is especially strongly associated with this period of time are often quite specific, mentioning persons, places, and events. This is consistent with the specificity found in MEAMs (for example, Schulkind et al., 1999; Janata et al., 2007). This specificity may be augmented by the well-documented negative emotions of the pandemic (e.g., Son et al., 2020; Wang et al., 2020; Xie et al., 2020; Copeland et al., 2021), as trauma and negative moods are linked to more imagery-filled memories (Wenzel et al., 2004), but not more fragmented or false memories (Storbeck and Clore, 2005; Kensinger and Schacter, 2006; Rubin et al., 2008).

Finally, across the 270 participants from both experiments, we collected 260 unique songs, many of which participants mentioned were discovered during the pandemic. These songs represent great diversity in music taste, as music listening is a subjective experience for each user and is influenced by factors such as personality and age (North, 2010). The participants reported using a number of Spotify features for discovering new music, especially in the period after lockdown. Those who used these features tended not to use features oriented toward rehearing favorite music. With over 60 million songs on demand (Spotify, 2020), and targeted song recommendations that aid listeners in finding new but familiar-feeling tracks (Luck, 2016), Spotify allows users the freedom to curate unique personal music repositories of old favorites and new finds.

Additionally, the results from our signature song analysis uncovered some of the central reasons that particular songs are especially associated with this period, and these reasons could be linked to lyrical content. In the emotion cluster, many lyrics were directly linked to emotion. Words such as “lonely” and “sad” are particularly interesting in the context of the pandemic, as individuals during this time feel especially lonely after long periods of social distancing (Xie et al., 2020), and as previously discussed, may use music as a tool to decrease this loneliness (Schäfer et al., 2020; Fink et al., 2021; Granot et al., 2021; Martín et al., 2021). In the memory cluster, there were many lyrics related to places, time, and people. As memories, especially autobiographical ones, involve retrieving episodes from one’s personal past, such words are particularly relevant. Finally, as expected due to the wide variety of songs that participants discovered during this period, the most frequent lyrics in the discover cluster seem less focused on a singular theme. Some of these phrases had a more positive tone, which may suggest that individuals are searching for songs that remind them of better days.

In sum, through quantitative measures of different music-listening contexts, as well as qualitative analyses of common themes in reasons for listening during this time, we document how undergraduates listen to and find music that is meaningful to them during the COVID-19 pandemic. The patterns found may stem from a combination of factors, such as changes in living situation, working from home, lack of social opportunities, and decreased mental wellbeing. Overall, it is remarkable that the results of these two experiments are similar despite their relocation to the university, likely reflecting the continued constraints imposed by the pandemic. Future research can expand on these findings by investigating whether the songs that participants deemed significant during this period will eventually evoke specific pandemic-related memories. It will be interesting to see whether the patterns of music listening from this period, together with many other behaviors, will persist in the future.

## Data Availability Statement

The raw data supporting the conclusions of this article will be made available by the authors, without undue reservation.

## Ethics Statement

The studies involving human participants were reviewed and approved by Institutional Review Board, Cornell University. The patients/participants provided their written informed consent to participate in this study.

## Author Contributions

All authors listed have made a substantial, direct and intellectual contribution to the work, and approved it for publication.

### Conflict of Interest

The authors declare that the research was conducted in the absence of any commercial or financial relationships that could be construed as a potential conflict of interest.

## Footnotes

^1^https://monkeylearn.com/word-cloud/

^2^https://genius.com/
